# Remarks on the Mortality of Children as the Result of Various Sources of Irritation from the First Dentition

**Published:** 1853-04

**Authors:** J. L. Levison


### ARTICLE II.

*Remarks on the Mortality of Children as the Result of Various Sources of Irritation from the First Dentition.* By J. L. Levison, D. D. S.

In the number of the Journal of Dental Science for October, (1852,) there was an article by V. M. Swayze, D. D. S. "On convulsions cured by lancing the gums," the author concluding his remarks with the following startling and important questions, "*What causes the death of ten thousand children annually in the city of New York? Has not* (he adds) *morbid dentition something to do with this fearful mortality.*"

Permit me, therefore, to offer some few observations on this interesting subject, as the result of much experience. Promising that we must in the investigation of diseases, as in the physical sciences, collect in the first instance facts, and then on the Baconian principle of induction, endeavor to trace the cause, or causes of the phenomena.

In the present instance to do this on sound *data,* we must constantly keep before us that the sources of irritation from teething, arises from the pressure of the developing teeth; and which in consequence implicate three important systems, viz. the NERVOUS, the VASCULAR, and the MUCOUS.*

1. THE NERVOUS.—That the nervous system is affected in dentition is proved by numerous recorded cases. But for my present purpose, it is important to state, that the convulsions from teething are not confined to the twitchings of the facial nerves, but in many instances there is involved, by reflex nervous action, the cerebral and spinal systems, as the following cases, among many others which I have noted down, will confirm.

During my residence at Hull, in Yorkshire, occasionally some professional engagements led me, from time to time, to visit Louth, a beautiful town in Lincolnshire, the inhabitants of which were, many of them, independent people, so that during my brief sojourn I had always plenty of occupation—particularly as my practice embraced many of the surrounding towns. It was on one of my visits, that Mrs. S—— called to ask me to see her little nephew, whom she described to be about one-and-twenty months old—that he had not a single tooth through his gums, that he had most awful fits, and that with all their care and watchfulness they could not prevent them; that they came on at all times, occasionally remaining for hours, and that during the fit his body was stiff, resembling one already dead. That it was frightful to see him, as his eyes seemed as if they would start from their sockets, that he had not any power to move them, and that when so conditioned, he had not any volition over the sphincters of the rectum or bladder, and she concluded by saying—"will you see him, for he is altogether a most awful looking being?"

Having ascertained that a medical man was attending him, and that her relatives resided eight miles in the country, I declined doing so, but from my personal friendship for the lady, I said "*his gums ought to be lanced.*" They have been, was her reply. Not sufficiently so, I remarked, or else the symp-

* I shall, if there is time and space, also incidentally show, that under certain circumstances, the *dermoid* tissues are sympathetically affected.

toms would be mitigated. That same day she drove into the country and reported our conversation to her relatives. The next morning while at breakfast a carriage stopped at my lodgings, and to my surprise Mrs. S——, accompanied by a servant carrying her nephew, were ushered into my room; and whilst the lady was apologising for, in a measure of forcing me to see the child, a fit came on, which was indeed most frightful. The *tetanic* state of the muscles—the eyes thrust forward without either motion or expression—the involuntary action of the rectum and the bladder—the deadly bluish hue of the skin, made altogether such a painful spectacle, which with the urgent entreaties of the aunt to do something, that however desirous of obeying professional usages, my humanity could not resist the appeal—so taking the boy, I said, "my dear madam as soon as the lancet touches the first tooth the fit will cease, and though it is my intention to cut down on every other tooth on both the jaws, the young gentleman may scream most lustily, (for he was a powerful made child,) but he will never have any repetition of the convulsions or the loss of consciousness." The result was as I had predicted. And the fit, which had hitherto lasted for hours, was banished as if by magic in a few seconds by the potency of a good incision, whose mandate was distinctly expressed as the lancet grated on the tooth.

[The prejudice against lancing the gums in teething, must have resulted in the practice of making merely a superficial incision. The late Dr. Davis, (professor of midwifery at the London University,) in his lectures on the diseases of children, recommended that a portion of the gum should be *nicked* out, so as to remove the resisting superincumbent surface, but I have found that feeling the tooth is all sufficient.]

It may be remarked that the gums were broad and full, and highly vascular, and as every tooth was relieved from its state of durance, there never occurred any return of the fits. But he was about ten or twelve years old when I last saw him—he appeared then to have a heaviness about the brow, dull staring projecting eyes, and a lack of intelligence in his expression, although the anterior lobes were good, which together gave presumptive evidence, that although my operation had been beneficial, yet he had prior to it, received some cerebral injury, but not to such a degree as to prevent his acquiring the rudiments of knowledge. However, this and similar cases show that irritation of the fibrils of the fifth pair of nerves, (the maxillary branch,) may by reflex action involve the brain and spinal nerves in their abnormal condition.

As an important physiological fact, I may add, that in the majority of such severe cases as detailed above, the sufferers have been of the nervo-bilious temperament. In my own practice I invariably cut down on a tooth whenever it is causing such local disturbance, as sleeplessness attended with constant moaning, even should there not be any symptom of nervous or cerebral irritation. This plan is most conservative. For it must be the soundest rule in practice, to prevent rather than permit a form of disease to set up, and then attempt to cure it.

It is admitted by most writers on the diseases of children, that hydrocephatic tendencies are often induced during teething, and the subject is often treated under two divisions, HYDROCEPHALOUS EXTERNUS, and HYDROCEPHALOUS INTERNUS. There is marked differences in the physiognomical expression in the two forms of the disease.* As it is the latter with which we have to do, as specially connected with our subject, being in most instances traceable to the first dentition. I select one case to illustrate this statement.

Mr. and Mrs. —— were highly intelligent persons, in a respectable position, but of highly nervous temperaments. As they had on many previous occasions lost their children during the teething, when Mrs. —— became pregnant she was very anxious and greatly excited about preserving the anticipated stranger. Yet "not more true than strange," they kept a great deal of company, setting up very late, the very reverse method of soothing her irritable nervous system. However, in the course of time, she gave birth to a very beautiful and fine boy,

* Hydrocephalous externus is often the form of the disease from which the offspring of drunkards suffer.

with a noble proportioned head, fine muscular limbs, and every appearance of good stamina.

The child improvd daily. Took notice of every thing, and was so much affected

"With the harmony of sweet sounds,"

as almost to jump out the person's arms holding him.

And, although I often protested against such injudicious treatment, they did not heed my friendly precautions not to over-stimulate his brain.

About the sixth month he had two lower teeth, which caused much disturbance to his health, as his pulse with quick and certain feverish symptoms. I urged the use of the lancet, and the child was relieved of the worst form; and they had on other occasions recourse to my services with evident advantage to the dear little boy.* When he was about nine or ten months old, having called on his parents, I saw symptoms of *strabismus* in the little fellow, and pointed out the appearance of his eyes to his mother, urging her to leave the room that I might lance his gums and save him from worse consequences. But she peremptorily refused, "as the child was feverish," &c. Within a few days acute symptoms of cerebral disease were indicated, (from the then distorted squint,) and when her usual attendant was summoned, he admitted that there existed *hydrocephalous internus* and although he adopted an active practice, yet nevertheless this lovely boy died within a week of my first seeing him.

Time rolled on, and the lady became again a mother, having had a fine and splendid girl born, but she told me that both herself and husband were melancholy, as Dr. —— had said they would never rear it. For that all their children would have the same form of cerebral disease that their last boy died of, and to this I ventured to a counter statement "that if the lancet had been freely used even in the case of their last child

*I may in this place observe, that being conscious not only of the suffering that children must feel in dentition, and the mortality thus induced, that I have made it my tribute to humanity to lance the gums for children brought to me, with very few exceptions, gratis.

at the period when I had first pointed out the pressure on the brain."

In his case the predisposition may have been induced by the late habits and great excitement during pregnancy—and by his brain having been over stimulated from birth. Without these disadvantages, he would in all human probability have yet existed, and I added, therefore, now act upon your past experience, let the mouth of the baby be attended to; be careful in her diet, in quantity and quality, and you will in all probability preserve your beautiful child to be a source of happiness to you. Facts proved the correctness of this view, and so impressed was the mother with my opinion, that she invariably sent to me to know "whether the child might go out—or if I thought the wind in a healthy quarter," &c.

The little Miss ——, though an excitable creature, grew well and strong, and is now a very fine intelligent girl, with a nice younger sister as a companion. But I may incidentally remark, that this child suffered great disturbance of health during the first dentition; and if the effects had not been so constantly counteracted by the *hydrag. cum. creta.* and the free use of the lancet, there is not a doubt but that she would have gone "to that bourne whence no traveller returns," and the opinion of hopelessness in the parents would have been established, that owing to some mysterious hereditary tendency, or some apparent want of *stamina*, they would never rear a family. I have repeatedly been requested to lance the gums of a family whose father had epileptic fits, with strong maniacal and hysterical symptoms. The mother was a most sensible woman, and acted under my instructions, and had never suffered any symptoms of dental irritation to pass, (except with one of her children,) without having the lancet freely used. In the case in which from some inadvertency this had been omitted, the child had severe fits; but even in the latter instance, the lancet saved a repetition of the fits, as the experience of the mother confirming my own, made her anxious never to suffer the slightest symptoms afterwards to be unheeded.

THE VASCULAR SYSTEM.—Under the head of vascular, we might mention fevers which are often induced by dentition whenever the teeth are evolved irregularly. When this vascular disturbance is confined to the buccal cavity, the inflammation is often acute, and ultimately induces a suppurative process.

Mrs. C——, a friend of mine, asked me to go into her nursery to see the baby. He was a most beautiful little boy, about a year old, with most expressive features, high and narrow forehead, with bright and intelligent eyes—but he was screaming most bitterly. And the short time I was occupied in asking a few questions, his shrieks were, every now and then, most piercing, that I could have cried myself in sympathy for the dear little fellow. On looking into his mouth, there was evidence of suppuration over one of the lower molars, and the area which covered the incisors had the blood vessels so much injected as to form a distinct boundary space between the vascular surface and the portion which indicated a normal condition. I freely lanced the affected gums, and scarified the vessel superficially. On opening the abscess in the region of the molar, nearly a tea-spoonful of yellow pus escaped. The child was so much benefited, and so immediately easy, that he soon fell into a sound sleep, and on awakening took some food with something like a sense of enjoyment, and by the next day his cheerfulness was restored, and the nursery rang with his merry laugh—

> "That the very air was musical
> With his shouts of warm delight."

It was my intention to have introduced the following case under the section of nervous disturbance, but I reserved it to show how one form of diseased action may be followed by others even when there exists but one idiopathic cause.

Mrs. C——, (the lady last mentioned,) had on a previous occasion solicited my services, and the recollection of which had no doubt induced her to be excited about the boy. It was some few years before that her eldest daughter had had fits, when not quite two years old, and was suffering from the last molars of both the upper and the lower jaws.

As there is matter for reflection in this case, I will give a summary of it: Little Miss C—— was, at the time I saw her, under the care of the celebrated Dr. R——— of London, who treated his patient as suffering from ingestia or worms in the alimentary canal, and he prescribed calomel powders. The third fit had taken place when I accidentally called to pay a hurried and friendly visit, when the anxious mother, (as there had been cases of cerebral disease in the family,) urged me to see the child. She did not say that any professional man was attending the little patient. On our entering the nursery, the dear little girl put her fingers into her mouth, and between a cry and a groan, said in her unintelligible language, "Da! Da!" which was evidently to tell me she was suffering in her mouth. On looking into it, I found it hot and vascular, and in particular over the still fast-bound teeth—the gums over which were swollen, and these I freely lanced, feeling the deep-seated teeth. And so conscious was the little lady of the benefit, that she put up her mouth to kiss me.

The true source of the sympathetic disturbance of the brain, (which having been in all probability hereditarily predisposed to be irritable,) was induced by the constant pressure of the teeth on the nervo-vascular mass, as the proximate cause, for when these were relieved, she never had another fit.

If, therefore, such an able practitioner as Dr. R———, one devoted to treat diseases of women and children, a very clever and learned man, and the author of a work on this special branch of practice—*if he omitted to look into the mouth of his suffering little patient, can Dr. Swayze wonder that there should be such mortality among children!*

The Mucous System.—Ever since Mr. Goodsir, of Edinburgh, had so ably demonstrated that the germs or rudimentary teeth were mucous follicles, practitioners have turned to account this important fact, viz. that when in teething there is severe attacks of diarrhea, that this arises merely from sympathetic disturbance of the mucous surfaces of the whole or part of the intestinal canal, and is therefore a consequence of dental irritation. The following will illustrate this theory: Mr. and Mrs. S———, of New York, visited England during the summer of 1852, with two little daughters; the eldest about fifteen or sixteen months old, and was suffering from tardy dentition

When they came to Brighton, this child was much reduced with constant watery and mucous evacuations. A London practitioner had given her a chalk mixture. When I saw her, she was very thin, very fretful, and highly irritable; without any appetite and with a furred tongue; and appeared from these united causes to be likely to sink from mere exhaustion. On examining her mouth, I told Mrs. S——— that all this disturbance of the health was occasioned by her teeth, and that if the gums were well lanced she would be physically and mentally better. And she, being a very sensible lady, immediately requested me to operate, which I did effectually. The diarrhea ceased—the secretions soon became normal—her appetite returned, and she became cheerful and daily gathered strength. But the disturbing cause had existed so long, that it was weeks before she entirely recovered.

I am tempted to quote another case: Master N———, a boy about twenty-one months old, gradually sunk. His body wasted, and his pulse was so feeble that he lay for hours in a state of *coma*, with the breathing scarcely apparent. When I was asked to see him, he seemed in *articulo-mortis*, so little did he resemble a being endowed with life. His face was an ashy whiteness—the eye-lids were closed—the extremities were cold, and the whole body felt clammy. He neither moved, nor gave any positive signs of animation. To my inquiries about his teeth, I was told that he had only four or five, that his illness commenced with pain and heat in the mouth, that Dr. ——— had lanced his gums, and that though somewhat better afterwards, that he gradually sunk and was a mere living skeleton.

As my own opinion still was that all the symptoms resulted from dental disturbance; arising in the first instance from the debility of his system, which latter circumstance had retarded the development of the teeth; and in consequence of this a still further disturbance was induced, affecting the brain and spinal nerves, and indirectly tended to implicate the organs of nutrition. As there seemed to the suffering parents no chance of their child being saved, they placed him entirely under my treatment, without even a forlorn hope that any advantage would result.

I took the poor emaciated creature across my lap and opened his mouth. There was a great fester, the gums being thick, red and swollen. I used the lancet freely, feeling every tooth which required this important aid. The child opened his eyes and smiled faintly. In an hour or two afterwards he was sufficiently restored to take some light nutritious food with an apparent relish. After this he rapidly recovered and is now strong and healthy, having, when I saw him last, attained the age of five years.

Thus it will be evident that at the period of teething, the brain and spinal nerves may be sympathetically implicated, and by reflex nervous action—the circulatory, the secretory and the assimulating organs are often involved; and sometimes there is an abnormal state of the external senses. That from the direct effect on the mucous surfaces, the abdominal organs suffer. Whilst the glands, the neck, the throat and the mouth generally may also become seriously affected. As a proof that the external senses may be implicated, we may refer to *strabismus*, and the deafness (which leaves a child a mute) arises in a majority of cases during dentition—the inflammatory condition of the mouth affecting the eustachian tube, and in consequence the child is deprived of the power of hearing articulated sounds, and never attempting to use his own vocal organs, hence he becomes a dumb mute.

Lastly, we may mention, that many *dermoid* affections are referrible to the same cause.* The law, seemingly is, that the weakest organs are likely to be first implicated; and that those, therefore, which we inherit from birth, with an hereditary tendency to diseased action, are thus certain of suffering the soonest from any violent disturbing influence. In selecting, therefore, the few cases now recorded from numerous others equally illustrative of the effects of early dentition, we think that they furnish an answer to Dr. Swayze's question, not only as regards the mortality of children of New York, but of all other

*As this paper is longer than I had anticipated, I must furnish in a future journal some interesting facts on the truth of this statement.

civilized cities. *For the loss of life is greatest from birth to two years or two years and a half old; the very period in which nature matures and develops the deciduous set of teeth.*

14 DEVONSHIRE PLACE, BRIGHTON, Feb. 1st, 1853.

